# Temporal trend of cancer mortality in a Brazilian state with a medium Human Development Index (1980–2018)

**DOI:** 10.1038/s41598-020-78381-4

**Published:** 2020-12-07

**Authors:** Marcela Sampaio Lima, Hianga Fayssa Fernandes Siqueira, Alex Rodrigues Moura, Evânia Curvelo Hora, Hugo Leite de Farias Brito, Adriane Dórea Marques, Érika de Abreu Costa Brito, Rosana Cipolotti, Carlos Anselmo Lima

**Affiliations:** 1 Health Sciences Graduate Program/Federal University of Sergipe, Aracaju, Brazil; 2 University Hospital/EBSERH/Federal University of Sergipe, Aracaju, SE 49060-108 Brazil; 3 College of Medicine/Federal University of Vale do São Francisco, Paulo Afonso, Brazil; 4 Department of Medicine/Federal University of Sergipe, Aracaju, Brazil; 5 Aracaju Cancer Registry, Aracaju, Brazil; 6 Call MS/CNPq/FAPITEC/SE/SES - No 06/2018, Fundação de Apoio à Pesquisa e à Inovação Tecnológica do Estado de Sergipe - FAPITEC/SE, Aracaju, Brazil

**Keywords:** Cancer, Medical research, Oncology

## Abstract

Emerging economy countries in epidemiological transition have been especially challenged in the fight against cancer. This was an ecological study that aimed to describe the temporal trend of cancer mortality in a Brazilian northeastern state with a medium Human Development Index using official Brazilian mortality data from 1980 to 2018. We calculated the mortality crude rate (CR) and age-standardized rate (ASR) based on official population counts and estimates. The Joinpoint Regression Program, National Cancer Institute, USA, was used to calculate time trends of cancer mortality. There were 34,214 deaths from cancer, excluding nonmelanoma skin cancer, in Sergipe. The overall cancer mortality ASR was 70.1 and 57.9 per 100,000 men and women, respectively. For the last five years, the leading causes of cancer deaths were prostate (21.3), trachea, bronchus and lung (11.7), stomach (6.5), oral cavity (5.4) and liver and intrahepatic bile ducts (5.1) in males and breast (13.8), trachea, bronchus and lung (6.6), cervix (6.4), colon/rectum (5.8) and central nervous system (3.6) in females. In addition, there was a significant reduction in deaths from ill-defined causes in the series. Our results show that although there has been an increase in cancer mortality rates associated with Western lifestyles, such as prostate, breast and colon/rectum, high rates of cancer related to poverty and infections, such as stomach and cervix, still persist in Sergipe.

## Introduction

Cancer is a major health problem worldwide and has a considerable impact on the population and health systems, especially in countries with emerging economies facing epidemiological transition^1,2^, such as Brazil.

According to GLOBOCAN 2018 estimates, there were 18.1 million new cases of cancer (17.0 million excluding nonmelanoma skin cancer—NMSC) and 9.6 million cancer deaths (9.5 million excluding NMSC) in 2018^1^. There has been a global decrease in cancer mortality rates in recent years, although it is less pronounced in underdeveloped regions, such as some Latin American countries. However, the total number of deaths is still rising, especially in emerging economy countries^1,3–5^.

The Brazilian National Cancer Institute (INCA) periodically publishes cancer incidence estimates of the nineteen main cancer sites in Brazil. For the 2020–2022 triennium, 625 000 new cases of cancer are expected each year (450 000 excluding NMSC). In 2018, there were 224,727 cancer-related deaths in Brazil, accounting for approximately 17% of the total deaths^6–8^.

There are some disparities in the incidence and mortality rates for specific types of cancer between the five geographic regions of Brazil, depending on the degree of social and economic development, exposure to risk factors and lifestyle. For example, in the Southeast Region, the epidemiological profile is similar to that in developed countries, while in the North and Northeast Regions, high rates of cancer incidence and mortality related to underdevelopment still persist^6^.

There are few studies on cancer incidence and mortality in Sergipe; existing studies are focused on a particular group or type of cancer^9–15^. This study aimed to describe the temporal trends of cancer mortality in Sergipe in a comprehensive way to compare the results with regions with similar HDI and signal priorities for actions against cancer. We also intended to assess the quality of official mortality data in our state.

## Methods

This was an exploratory ecological study of time trends. Data from 1980 to 2018 were obtained from the Mortality Information System (SIM database).

Sergipe is located on the coast of the Northeast Region of Brazil in the 24 south zone. It has a tropical climate, a population of 2,068,017 inhabitants (census 2010), an estimated population of 2,278,308 for 2018 and a Human Development Index (HDI) of 0.665 (census 2010). Only the capital, Aracaju, has a high HDI (0.77) and is home to approximately 28% of the state’s population, while the other 74 municipalities have medium or low HDI^16–18^.

We included individuals who died from cancer of any site or ill-defined site residing in the state of Sergipe. We calculated crude rates (CRs) and age-standardized rates (ASRs), adjusted by the world population^19,20^, for all ages and age groups by stage of life (0–19, 20–44, 45–64 and 65+) for both sexes and by the main sites of cancer, based on official population counts and estimates from the Brazilian Institute of Geography and Statistics (IBGE).

According to the primary site, the cases were classified and sometimes grouped based on the International Classification of Diseases, 10th edition (ICD-10), as follows: oral cavity—C00 to C10; esophagus—C15; stomach—C16; colon and rectum—C18 to C21; liver and intrahepatic bile ducts—C22; larynx—C32; trachea, bronchus and lung—C33 and C34; melanoma skin cancer—C43; nonmelanoma skin cancer—C44; female breast—C50; cervix uteri—C53; corpus uteri—C54; ovary—C56; prostate—C61; bladder—C67; central nervous system—C70 to C72; thyroid gland—C73; Hodgkin’s lymphoma—C81; non-Hodgkin’s lymphoma—C82 to C85 and C96; leukemias—C91 to C95. The other sites were grouped as ‘other’ and added to cancers of unspecified topography. For the temporal analysis of ill-defined causes of death, we selected cases with codes R69, R98 and R99 based on ICD-10.

The Joinpoint Regression Program, version 4.7.0.0, from the National Cancer Institute, USA^21^, was used to calculate time trends for age-standardized rates of cancer mortality using a model based on the assumption of a minimal number of join points where statistically significant changes in the curves occur. Additionally, the annual percent change (APC) and the average annual percent change (AAPC), which are the summary measures of the trends over the analyzed period, with their respective 95% confidence intervals (CI 95%) and p values, were calculated by the program. A significant change in a trend was defined as p < 0.05.

The present project was submitted to the Research and Ethics Committee of Federal University of Sergipe (*Universidade Federal de Sergipe—UFS*), and it was approved and registered under CAAE number 57995416.9.0000.5546. We declare that all methods were in agreement with the relevant guidelines and regulations. For the sake of confidentiality, we used deidentified databases. Therefore, it was not possible to obtain informed consent. The Research and Ethics Committee of the Federal University of Sergipe exempted informed consent, in agreement with Resolution 466 of December 2012 of the Ministry of Health, Brazil.

## Results

During the time series, there were 34,672 deaths from cancer in Sergipe (34,214 deaths excluding NMSC), accounting for a total of 9.27% of all deaths. There were 17,322 deaths in males and 17,350 in females. Excluding NMSC, the overall age-standardized mortality rate was 70.1/100,000 men and 57.9/100,000 women for the whole period. Table 1 shows the distribution of the cancer deaths, with the respective mortality CRs and ASRs, by the main sites for both sexes.

**Table 1:** Distribution of the number of cancer deaths (N) by the main sites, with respective age-standardized mortality rates (ASR) per 100,000 men and women and 95% confidence intervals (95% CI) in Sergipe, 1980–2018.

Sites	Male			Female		
	N	ASR	95% CI	N	ASR	95% CI
Oral cavity	878	3.8	3.51; 4.01	299	1.0	0.89; 1.11
Esophagus	709	3.0	2.82; 3.26	210	0.7	0.61; 0.81
Stomach	1339	5.7	5.37; 5.98	786	2.6	2.45; 2.82
Colon and rectum	698	2.9	2.71; 3.14	992	3.3	3.12; 3.54
Liver and intrahepatic bile ducts	965	4.1	3.82; 4.33	881	3.0	2.81; 3.21
Larynx	613	2.7	2.45; 2.88	93	0.3	0.26; 0.40
Trachea, bronchus and lung	2232	9.7	9.28; 10.08	1442	5.0	4.75; 5.26
Melanoma skin cancer	115	0.5	0.38; 0.55	94	0.3	0.24; 0.37
Prostate	3354	13.5	13.07; 13.98	–	–	–
Breast	–	–	–	2773	9.5	9.13; 9.84
Cervix	–	–	–	1925	6.6	6.26; 6.84
Corpus uteri	–	–	–	163	0.6	0.49; 0.67
Ovary	–	–	–	643	2.2	2.04; 2.38
Bladder	406	1.7	1.54; 1.87	158	0.5	0.44; 0.60
Central nervous system	723	2.7	2.49; 2.89	749	2.5	2.29; 2.65
Thyroid	47	0.2	0.14; 0.25	103	0.4	0.29; 0.43
Hodgkin’s lymphoma	90	0.3	0.24; 0.37	49	0.2	0.11; 0.20
Non-Hodgkin lymphoma	453	1.7	1.54; 1.85	329	1.1	0.95; 1.18
Leukemias	748	2.5	2.35; 2.71	655	2.0	1.86; 2.17
Other sites	3715	15.2	14.69; 15.67	4785	16.2	15.70; 16.62
Subtotal	17,085	70.1	69.06; 71.17	17,129	57.9	57.01; 58.75
Nonmelanoma skin cancer	237	1.0	0.83; 1.07	221	0.7	0.57; 0.74
Total	17,322	71.1	70.01; 72.13	17,350	58.5	57.66; 59.41

**Figure 1:**
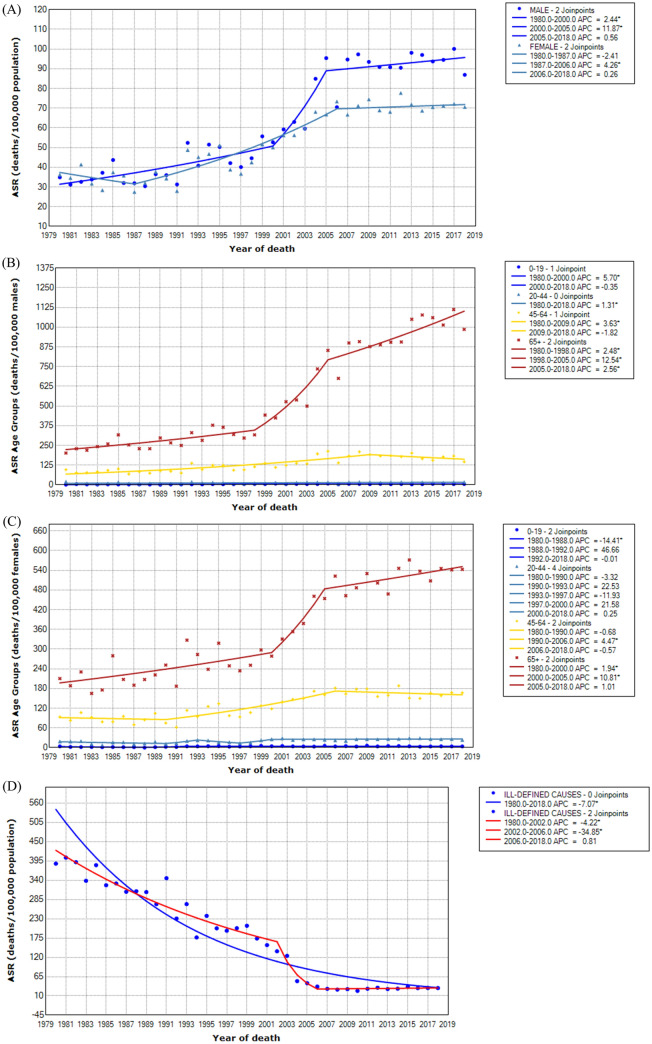
Time trends of cancer mortality for age-standardized rates (ASR) for all ages and sites in both sexes (**A**), for specific age groups in males (**B**) and in females (**C**) and for ill-defined causes of death (**D**).

Table 2 depicts the joinpoint analysis of cancer mortality by all ages and age groups for both sexes and for the ill-defined causes of death (male and female). For males, there was a statistically significant upward trend from 1980 to 2000 (APC 2.44) and from 2000 to 2005 (APC 11.87) and subsequent stabilization of the curve. For females, the curve was stable until 1987, and there was a statistically significant upward trend from 1987 to 2006 (APC 4.26) and new stabilization thereafter. The trend curve of the ill-defined causes of ASR showed a statistically significant decrease from the beginning of the series until 2006 and stabilized later. The curves modeled by Joinpoint are represented in Fig. 1.

**Table 2:** Joinpoint analysis of cancer mortality for age-standardized rates for all ages and specific for age groups for both sexes. Sergipe, 1980–2018.

Age groups	JP	APC	95% CI	AAPC	95% CI
**Male**					
All	2	(1980–2000) 2.44^a^	1.15; 3.74	2.98^a^	1.55; 4.42
	–	(2000–2005) 11.87^a^	1.91; 22.80		
	–	(2005–2018) 0.56	− 0.60; 1.74		
0–19	1	(1980–2000) 5.70^a^	3.11; 8.35	2.79^a^	1.17; 4.43
	–	(2000–2018) − 0.35	− 2.44; 1.78		
20–44	0	(1980–2018) 1.31^a^	0.53; 2.10	1.31^a^	0.53; 2.10
45–64	1	(1980–2009) 3.63^a^	2.78; 4.49	2.31^a^	1.43; 3.21
	–	(2009–2018) − 1.82	− 4.48; 0.91		
65+	2	(1980–1998) 2.48^a^	0.91; 4.06	4.29^a^	3.08; 5.51
	–	(1998–2005) 12.54^a^	7.13; 18.21		
	–	(2005–2018) 2.56^a^	1.51; 3.62		
**Female**					
All	2	(1980–1987) − 2.41	− 7.82; 3.31	1.73^a^	0.55; 2.93
	–	(1987–2006) 4.26^a^	3.24; 5.30		
	–	(2006–2018) 0.26	− 0.89; 1.42		
0–19	2	(1980–1988) − 14.41^a^	− 21.48; − 6.70	1.28	− 3.31; 6.09
	–	(1988–1992) 46.66	− 2.72; 121.09		
	–	(1992–2018) − 0.01	− 1.02; 1.01		
20–44	4	(1980–1990) − 3.32	− 7.58; 1.13	2.24	− 3.91; 8.79
	–	(1990–1993) 22.53	− 27.45; 106.93		
	–	(1993–1997) − 11.93	− 29.67; 10.28		
	–	(1997–2000) 21.58	− 21.34; 87.90		
	–	(2000–2018) 0.25	− 0.76; 1.28		
45–64	2	(1980–1990) − 0.68	− 4.39; 3.18	2.13^a^	0.57; 3.71
	–	(1990–2006) 4.47^a^	2.92; 6.06		
	–	(2006–2018) 0.57	− 1.91; 0.78		
65+	2	(1980–2000) 1.94^a^	0.76; 3.12	2.56^a^	1.65; 3.48
	–	(2000–2005) 10.81^a^	1.81; 20.61		
	–	(2005–2018) 1.01	− 0.04; 2.08		
**Ill-defined causes**					
Total	2	(1980–2002) − 4.22^a^	− 4.85; − 3.59	− 7.07^a^	− 8.04; − 6.09
	–	(2002–2006) − 34.85^a^	− 47.65; − 18.93		
	–	(2006–2018) 0.81	− 2.69; 4.44		

*JP* number of join points, *APC* annual percent change, *AAPC* average annual percent change, *CI* Confidence interval.

^a^APC/AAPC statistically significant.

Considering only the last five years of the series, which are the ones that best reflect the current scenario and projections, the mortality ASR was 97.2 and 72.0 per 100,000 men and women, respectively. For this period, the leading causes of cancer death were prostate, lung/trachea/bronchus, stomach, oral cavity and liver/intrahepatic bile ducts in males and breast, lung/trachea/bronchus, cervix, colon/rectum and central nervous system (CNS) in females (Table 3).

**Table 3:** Number of cases of the leading causes of cancer deaths (N), with respective age-standardized mortality rates (ASR) per 100,000 men and women and 95% confidence intervals (95% CI) in Sergipe, 2014–2018.

Male				Female			
Sites	N	ASR	95% CI	Sites	N	ASR	95% CI
Prostate	971	21.3	19.97; 22.65	Breast	853	13.8	12.90; 14.76
Lung/Tr/Br	529	11.7	10.67; 12.66	Lung/Tr/Br	396	6.6	5.92; 7.21
Stomach	304	6.5	5.80; 7.27	Cervix	391	6.4	5.73; 6.99
Oral cavity	261	5.4	4.78; 6.10	Colon/rectum	356	5.8	5.17; 6.37
Liver/IHBD	236	5.1	4.49; 5.80	CNS	211	3.6	3.15; 4.13
Total^a^	4552	97.2	94.42; 100.07	Total^a^	4408	72.0	69.91; 74.17

*ASR* age-standardized rate (world population), *CI* Confidence interval, *Br* bronchus, *Tr* trachea, *CNS* Central nervous system, *IHBD* Intrahepatic bile ducts.

^a^All cancer deaths, except NMSC.

**Figure 2:**
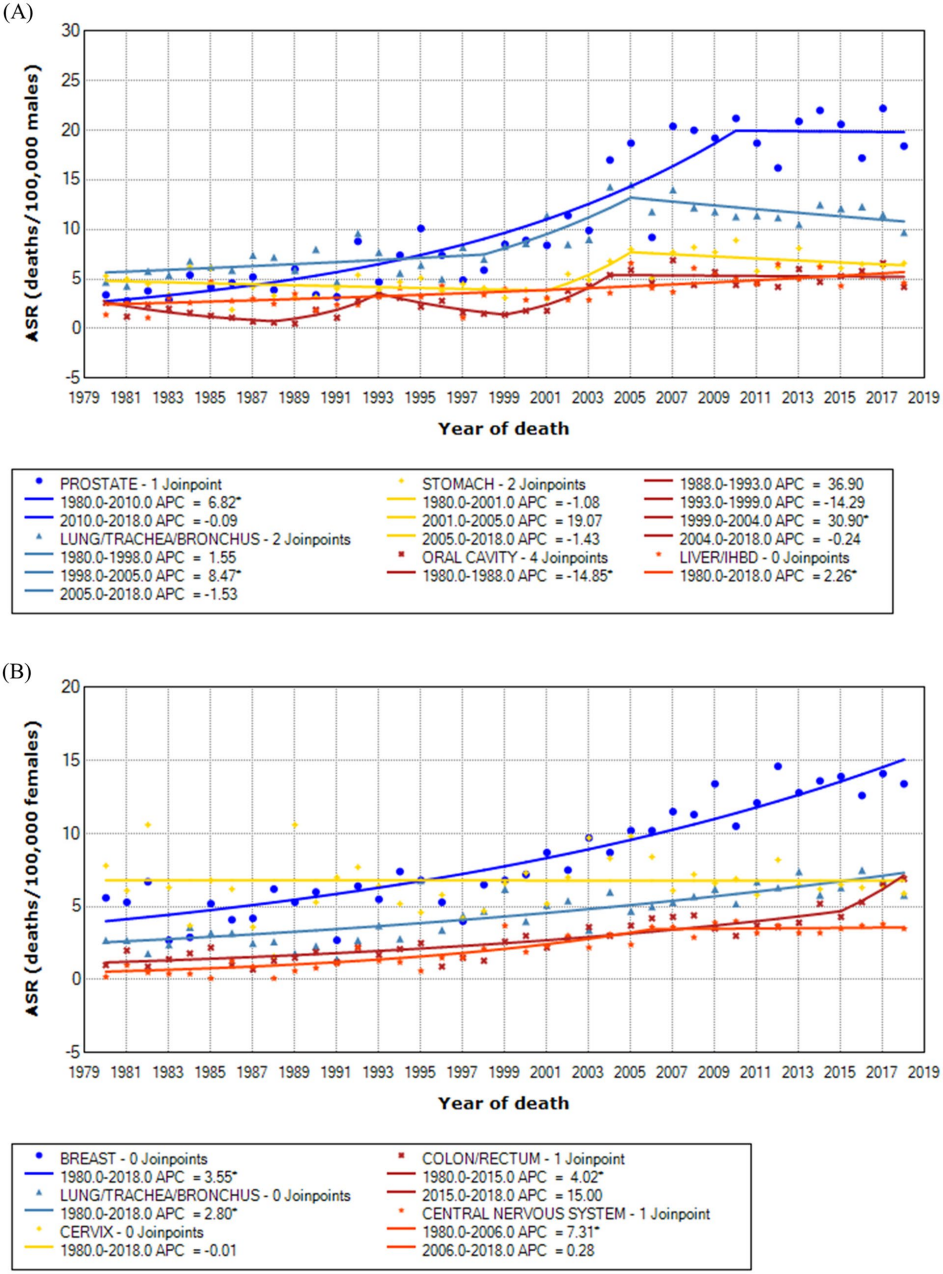
Time trends of cancer mortality for age-standardized rates (ASR) for the main sites in males (**A**) and females (**B**).

Table 4 shows the joinpoint analysis of cancer mortality by the main sites for both sexes. The curves modeled by Joinpoint are shown in Fig. 2.

**Table 4:** Joinpoint analysis of cancer mortality by the main sites for age-standardized rates for both sexes. Sergipe, 1980–2018.

Sites	JP	APC	95% CI	AAPC	95% CI
**Male**					
Prostate	1	(1980–2010) 6.82^a^	5.40; 8.25	5.32^a^	3.87; 6.80
	–	(2010–2018) − 0.09	− 4.66; 4.69		
Lung/trachea/bronchus	2	(1980–1998) 1.55	− 0.42; 3.57	1.72^a^	0.11; 3.35
	–	(1998–2005) 8.47^a^	1.37; 16.08		
	–	(2005–2018) − 1.53	− 3.16; 0.13		
Stomach	2	(1980–2001) − 1.08	− 2.89; 0.76	0.74	− 2.25; 3.83
	–	(2001–2005) 19.07	− 9.37; 56.42		
	–	(2005–2018) − 1.43	− 3.57; 0.75		
Oral cavity	4	(1980–1988) − 14.85^a^	− 26.35; − 1.55	1.79	− 5.17; 9.25
	–	(1988–1993) 36.90	− 4.90; 97.07		
	–	(1993–1999) − 14.29	− 31.03; 6.52		
	–	(1999–2004) 30.90^a^	2.20; 67.66		
	–	(2004–2018) − 0.24	− 2.33; 1,90		
Liver/IHBD	0	(1980–2018) 2.26^a^	1.46; 3.07	2.26^a^	1.46; 3.07
**Female**					
Breast	0	(1980–2018) 3.55^a^	3.01; 4.09	3.55^a^	3.01; 4.09
Lung/trachea/bronchus	0	(1980–2018) 2.80^a^	2.14; 3.48	2.80^a^	2.14; 3.48
Cervix	0	(1980–2018) − 0.01	− 0.76; 0.74	− 0.01	− 0.76; 0.74
Colon/rectum	1	(1980–2015) 4.02^a^	3.08; 4.98	4.85^a^	3.25; 6.48
	–	(2015–2018) 15.00	− 3.22; 36.65		
CNS	1	(1980–2006) 7.31^a^	5.02; 9.65	5.04^a^	3.31; 6.80
	–	(2006–2018) 0.28	− 2.51; 3.14		

*JP* number of join points, *APC* annual percent change, *AAPC* average annual percent change, *CI* Confidence interval, *CNS* central nervous system, *IHBD* intrahepatic bile ducts.

^a^APC/AAPC statistically significant.

## Discussion

The mortality curves for all cancers showed a significant increase until 2005 for men and 2006 for women and then they stabilized. At the same time, there was a significant drop in the mortality ASR from ill-defined causes. We believe that this is due to several factors: the implementation of the Unified Health System (SUS) in 1988 and family health programs, which made it possible for poor people to access health services, diagnostic resources and screening programs; government encouragement for health professionals to work in smaller cities and remote areas; greater coverage of SIM in the national territory with consequent reduction of underregistration deaths also in Sergipe^22,23^; and the improvement of the SIM database based on employee training in data coding.

According to GLOBOCAN 2018, mortality rates for all cancers combined worldwide are nearly 50% higher in males than in females^1^. We also verified a higher mortality ASR among males both in the entire series (70.1 versus 57.9) and in the last five years (97.2 versus 72.0). The average mortality ASRs from 2014 to 2018 were similar to those observed for the group of medium HDI countries in GLOBOCAN 2018^1^.

As expected for chronic diseases, the 65+ age group showed the greatest increase in the mortality trend, most pronouncedly among men. Interestingly, the male age group 20–44 years old presented a slight increasing trend during the entire series (APC/AAPC of 1.31), which likely reflects improved information on death certificates and decreased deaths from ill-defined causes. The curves for the other age groups remained stable for the past decade in both sexes.

In our study, lung cancer was the leading cause of cancer-related death for both sexes combined. It also remains the leading cause of cancer death worldwide, accounting for 18.4% of all cancer-related deaths, followed by colorectal (9.2%), stomach (8.2%), liver (8.2%) and breast (6.6%)^1^.

Among men only, prostate cancer was the main cause of death in our population, followed by lung/trachea/bronchus, stomach, oral cavity and liver/intrahepatic bile ducts. Among women only, breast cancer was the main cause of death in our population, followed by lung/trachea/bronchus, cervix, colorectal and CNS. This profile is similar to more developed countries where higher mortality rates from lung, breast and colorectal cancers are observed and it is also similar to less developed countries where high rates of cervix, stomach and oral cavity cancers are observed^1–5,24^.

In our population, there was a statistically significant increase in lung cancer mortality in the period from 1998 to 2005 among men (APC 8.47) with stabilization of the curve afterwards. Among women, there was a steady, but less dramatic, increase across the series (APC/AAPC 2.8), possibly reflecting a lower degree of exposure to risk factors. The average mortality ASRs in the last 5 years of analysis—11.7/100,000 men and 6.6/100,000 women—were similar to those observed in low/medium HDI countries^1^. Lung cancer incidence and mortality have changed substantially over time, expressing different trends by sex, age group and region throughout the world, according to historic patterns of smoking prevalence, air pollution and other risk factors^1,25^. Most developed countries have experienced periods of increased incidence and mortality, first among men and then among women. After a certain period, their rates started to stabilize and then decline, first among men and then among women, mainly due to the decrease in smoking prevalence across generations. More recently, medium HDI countries seem to follow this same pattern^3,4,24,25^.

Mortality from prostate cancer showed a statistically significant increase until 2010 (APC 6.82) and then it stabilized, reaching an ASR of 18.4 per 100,000 men in 2018 in our study. Lima et al., in a study on mortality from prostate cancer in Aracaju, also found an upward trend with a statistically significant APC of 2.1 and an average ASR of 23.2/100,000 men from 1996 to 2006^10^. Mortality rates from prostate cancer have been increasing in many countries in Central and South America (including Brazil) and in less developed countries in Asia and Europe. In contrast, mortality trends have stabilized or have been decreasing in recent years in the USA and other developed countries from Europe, Oceania and Asia, where early diagnosis appears to have played an important role^1,4,24^.

Breast cancer had the highest mortality ASR among our female population (13.8 in the last five years of the series), which is similar to the world media (13.0) and to trends observed in the Pan-American region^1,26^. In our study, the temporal trend continues rising throughout the series. We also observed that three age groups—20–44, 45–64 and 65+ years old—showed statistically significant upward trends throughout the series (data not shown). Breast cancer is the leading cause of cancer death among women in over 100 countries^1^. Martínez-Mesa et al. analyzed whether the HDI could explain differences in the incidence and mortality rates from breast cancer and gynecological cancers in the Pan-American region. They found that the HDI showed a positive correlation with breast cancer incidence and mortality rates and a negative correlation with cervical cancer incidence and mortality rates^26^.

In our study, the cervical cancer mortality ASR (6.4) was similar to that observed for the world (6.8); it was higher in relation to North America (2.6), but it was lower than that observed in South America (8.6), Central America (8.9) and the Caribbean (8.6)^26^. Although the temporal trend in mortality from cervical cancer was stable across the series in our state, we expect rates to decrease in the future after the addition of the tetravalent HPV vaccine to the national immunization calendar. The vaccination campaign started in 2014 for girls and in 2017 for boys. Currently, the target age group for vaccination is 9–14 years old^27^.

Colorectal cancer was the fourth leading cause of cancer death among women and the seventh among men in the period from 2014 to 2018. It presented a statistically significant upward trend curve with an AAPC of 4.85 and 3.96 across the series for females and males, respectively (data not shown). This means that this site could soon be among the top five for males in our state. The mortality rate for colorectal cancer has been increasing in Latin America, the Caribbean and South Africa, while it has been decreasing in most developed countries from Northern and Western Europe, North America and Oceania^1,3,4,28^. It is the third leading cause of death among men and women, separately, in the United States and the second and third leading causes of death in men and women, respectively, in Europe^4,24^. We believe that screening strategies, such as fecal occult blood tests or even colonoscopy, should be implemented for specific age groups to decrease mortality.

Stomach cancer was the third cause of cancer mortality in our male population, and it showed a stationary curve across the series. Since *Helicobacter pylori* causes approximately 75% of noncardia carcinomas of the stomach^3,29^, we assume that the prevalence of *H. pylori* among men is still high in Sergipe. There was a decrease in the mortality of stomach cancer in some countries in Asia (from 2000 to 2010)^3^ and in most countries in Latin America mainly thanks to improvements in basic sanitation, food preservation, health surveillance actions and the control of *H. pylori* infection. Carioli et al. pointed out that although there was a decrease in stomach cancer mortality from 1980 to 2014, rates still remained high in Latin America, particularly in Chile^5^.

According to the INCA protocol, the following sites were grouped as oral cavity: lips, oral cavity, salivary glands and oropharynx (C00–C10)^6^. The most well-known risk factors for these cancers are smoking, heavy alcohol intake, unprotected sun exposure (for lip cancer), HPV infection (especially for oropharynx cancer) and obesity^6,30^. Oral cavity cancer was the fourth leading cause of cancer death among men in our study (ASR of 5.4 in the last 5 years), but it showed a steady trend over the past decade. This mortality ASR was similar to the average observed among countries with low/medium HDI for men (5.0) and higher than the average observed for countries with high/very high HDI (1.5), according to GLOBOCAN 2018. Moreover, this cancer group was not among the main causes of cancer death among women, as observed for other regions of the world, regardless of the HDI^1^. Miranda-Filho and Bray pointed out that both incidence and mortality rates from oral cavity cancer were consistently higher among males than females globally, mainly reflecting the level of exposure to risk factors^30^.

For liver/intrahepatic bile duct mortality, we observed an upward trend with a statistically significant APC of 2.26 among men in Sergipe. According to GLOBOCAN 2018, mortality rates were 2 to 3 times higher among men in most world regions^1^. Hashim et al. also observed an upward trend in liver cancer mortality in North America, most Latin America and some Asian countries^3^. We emphasize that some of our cases correspond to metastatic carcinomas of undetermined primary sites. This limited our analysis on the role of hepatitis B and C virus infections in the onset of primary liver cancer.

In our study, the CNS was the fifth main site of cancer mortality among women and showed a stationary trend in the past decade. Like the liver, the CNS is a frequent site of metastasis. As we did not have access to histopathological data on patients, our analysis of primary CNS cancer mortality was limited.

As a strength of our study, we highlight that it presents cancer mortality over a long time period, and it was based on official data. Furthermore, there has been a progressive improvement in quality data of death certificates and in SIM coverage in our state, which can be verified by the progressive decrease in the mortality rates from ill-defined/undetermined causes since the beginning of the series in Sergipe.

Our study has some limitations. We did not include race in the analysis because we think this information is not reliable in our population. There is a high degree of miscegenation in Brazil, especially in the northeastern region. Furthermore, the race classification is based on how the individual self-reports, usually reflecting some social prejudices. We also did not have access to patients’ medical records, including the date of diagnosis, histopathological reports, treatments performed or hospitalizations. Further studies may go deeper into those topics.

## Conclusion

Our results show that although there has been a statistically significant increase in cancer mortality rates associated with Western lifestyles, such as prostate, breast and colon/rectum (especially in women), high rates of cancer related to poverty and infections, such as stomach, cervix and oral cavity, still persist. This profile is similar to that observed in regions that have low and medium HDI and that are usually facing epidemiological transition.

In addition, due to population aging, we expect cancer mortality to rise in our state. Therefore, the government should implement or emphasize some measures and control strategies for the prevention of the most common types of cancer, which are evidence-based and well-established in developed countries, such as tobacco-control policies, vaccination programs (e.g., HPV and hepatitis B viruses), treatment for *H. pylori*, screening programs such as Papanicolau tests for cervical cancer and mammography and encouraging a healthier lifestyle.

We hope that this brief review of the epidemiology of mortality for the main sites of cancer in a low/medium HDI region will contribute to global findings. We also emphasize the importance of having high quality national databases with wide coverage so that they reflect real-world situations in the best possible way.
